# Functional Anatomy and Clinical Significance of the Lateral Ankle Ligaments: A Cadaveric Study

**DOI:** 10.7759/cureus.109803

**Published:** 2026-05-28

**Authors:** Ajay K Panda, Wilmer Rodriguez, Keenan Horani, Vinod Panchbhavi

**Affiliations:** 1 John Sealy School of Medicine, University of Texas Medical Branch, Galveston, USA; 2 Department of Orthopedic Surgery and Rehabilitation, University of Texas Medical Branch, Galveston, USA

**Keywords:** anatomic variation, anatomy, ankle injuries, ankle sprains, anterior tibiofibular ligament, calcaneofibular ligament, lateral ankle ligament complex, posterior tibiofibular ligament

## Abstract

Introduction: Tears in the lateral ankle ligaments are among the most common injuries in both athletes and the general population. The ligaments involved in the lateral ankle complex include the anterior tibiofibular ligament (ATFL), calcaneofibular ligament (CFL), and posterior tibiofibular ligament (PTFL). Information on the anatomy and variation of these structures is currently limited. This study aims to provide detailed documentation of the anatomy and morphometry of the lateral ankle ligament complex (LALC).

Methods: This study involved 22 cadaveric ankles. Ankles with pre- or post-mortem injuries or prior surgical interventions were excluded. The ligaments were exposed following detailed dissection of the anterior and lateral aspects of the ankle, according to Cunningham’s dissection manual. Measurements of length and width were taken using a digital vernier caliper, and morphological characteristics, including shape and orientation, were recorded.

Results: The ATFL was noted to have four different shape morphologies, including single-banded (14/22 = 64%), double-banded (4/22 = 18%), V-shaped (3/22 = 14%), and Y-shaped (1/22 = 4.5%). The ATFL had an average length of 23.8 mm and an average width of 11.4 mm. The CFL had no variation in shape and was only seen in single-banded formation; it had an average length of 22.9 mm and an average width of 7.8 mm. The PTFL had no variation in shape and was only seen in single-banded formation; it had an average length of 18.1 mm and an average width of 7.8 mm.

Discussion: Our investigation highlights the variability of the LALC in both size and shape. Understanding the anatomical characterization of these ligaments is crucial for orthopedic surgeons when planning repair or reconstruction.

## Introduction

Lateral ankle injuries, which involve tearing one or more of the lateral ankle ligaments, are among the most common injuries in both athletes and the general public. Approximately three million ankles are sprained in the United States annually, which equates to an incidence rate of approximately 2.15 sprains per 1,000 individuals [1]. While the majority of these cases can be managed nonoperatively, approximately 1% to 5% will require some sort of surgical stabilization [2]. The ligaments involved in the lateral ankle complex include the anterior tibiofibular ligament (ATFL), calcaneofibular ligament (CFL), and posterior tibiofibular ligament (PTFL). Tears of the ATFL are the most common, accounting for approximately 65% of lateral ankle injuries [3,4]. The combined ATFL and CFL ruptures account for another 20% to 30% of lateral ankle injuries. The PTFL is the strongest of the three and is the least commonly injured [5].

Surgical treatment is reserved for patients after more conservative options such as rest and physical therapy have failed, have a high recurrence of ankle sprains, or have developed chronic lateral ankle instability [2,6]. The Broström-Gould technique, introduced in the 1980s, is a common surgical approach for this injury pattern and has generally been considered the gold standard in treatment [7]. Other options, such as open or reconstructive autografts using the extensor retinaculum and allografts, have shown similar outcomes [8]. The Broström-Gould technique, however, is associated with complication rates ranging from approximately 6% to as high as 30%. These complications most commonly include persistent instability, nerve irritation, traumatic retear, and postoperative stiffness [9-11]. These complications can be partially attributed to the fact that arthroscopic Broström repairs primarily address the superior (intra-articular) ATFL fibers and are most effective when sufficient ligament tissue remains for direct repair [12]. When the ATFL is severely attenuated or absent, arthroscopic repair may be insufficient, and reconstructive procedures provide better mid-term outcomes in terms of joint stability and functional outcomes [13].

Anatomical variations in size, shape, and quality of these ligaments are one proposed mechanism that may contribute to chronic pain following surgery [2,14]. Biomechanical evidence demonstrates that anatomic repair or reconstruction of both the ATFL and CFL is superior to nonanatomic techniques in restoring ankle stability and normal joint kinematics, minimizing the risk of chronic pain and osteoarthritis [15-17]. The importance of addressing anatomical variation is further underscored by findings that augmented ATFL repair with CFL repair best restores anterior translation and talar tilt stability, and that combined repair optimizes load transfer across midfoot and hindfoot joints [15-18]. Despite advances in imaging and surgical technique, the anatomical characterization of the lateral ankle ligaments remains limited in the literature.

This study aims to provide detailed documentation of the anatomy and morphometry of the lateral ankle ligament complex (LALC), including the ATFL, PTFL, and CFL. We hypothesized that we would identify greater variation in the LALC, including variants not currently described in standard anatomy textbooks. By characterizing these variations, we hope to inform surgical decision-making when addressing LALC-related fixation and improve outcomes for patients with chronic lateral ankle instability.

## Materials and methods

Twenty-eight formalin-fixed cadaveric specimens donated to the University of Texas Medical Branch gross anatomy laboratory were used in this study. Cadavers with a documented history of limb injuries, visible scarring around the ankle region, prior ankle fractures, or ankle surgeries were excluded, leaving 22 individual ankles that were dissected for analysis. None of the ankles used in this study had a corresponding contralateral ankle, as the cadaver specimens were obtained from the teaching lab and only one lower extremity per cadaver was preserved for this study. Dissections were done by authors A.K.P. and W.R. under the guidance of senior authors K.H. and V.P.. Ankles were placed laterally on the dissection table with the foot held in a neutral position. The dissection followed the procedures outlined in Cunningham's dissection manual, which prescribes a systematic layer-by-layer dissection technique used to expose the ligamentous architecture surrounding the talocrural and subtalar joints [19]. This process was done by first carefully making a horizontal incision across the lateral ankle and reflecting the skin, then dissection through the connective tissue, musculature, tendons, and neurovascular elements to reveal the level of the ATFL, PTFL, and CFL in their anatomical locations at the lateral ankle joint. Using a scalpel, any excess connective tissue on the insertion and origin points of the ligament was cleaned off to clearly identify ligamentous fibers for accurate measurement and morphological analysis.

Measurements of length and width were taken using a digital vernier caliper, and morphological characteristics, including shape and orientation, were recorded. Ligament length was measured with a caliper from the center of the insertion point to the center of the origin point. Ligament width was measured with a caliper and assessed by measuring the widest point of each ligament. Measurements were recorded in millimeters to the nearest 10th decimal point. The morphological shape of the ligaments was classified based on the direction of their fibers being either single-banded, double-banded, parallel, V-shaped, or Y-shaped. To minimize observer bias, all measurements were independently performed by three authors, A.K.P., W.R., and K.H.. Mean and standard deviation values were then calculated and recorded for each measurement using Microsoft Excel Version 16.101.3 (Microsoft Corp., Redmond, WA, USA).

## Results

**Figure 1:**
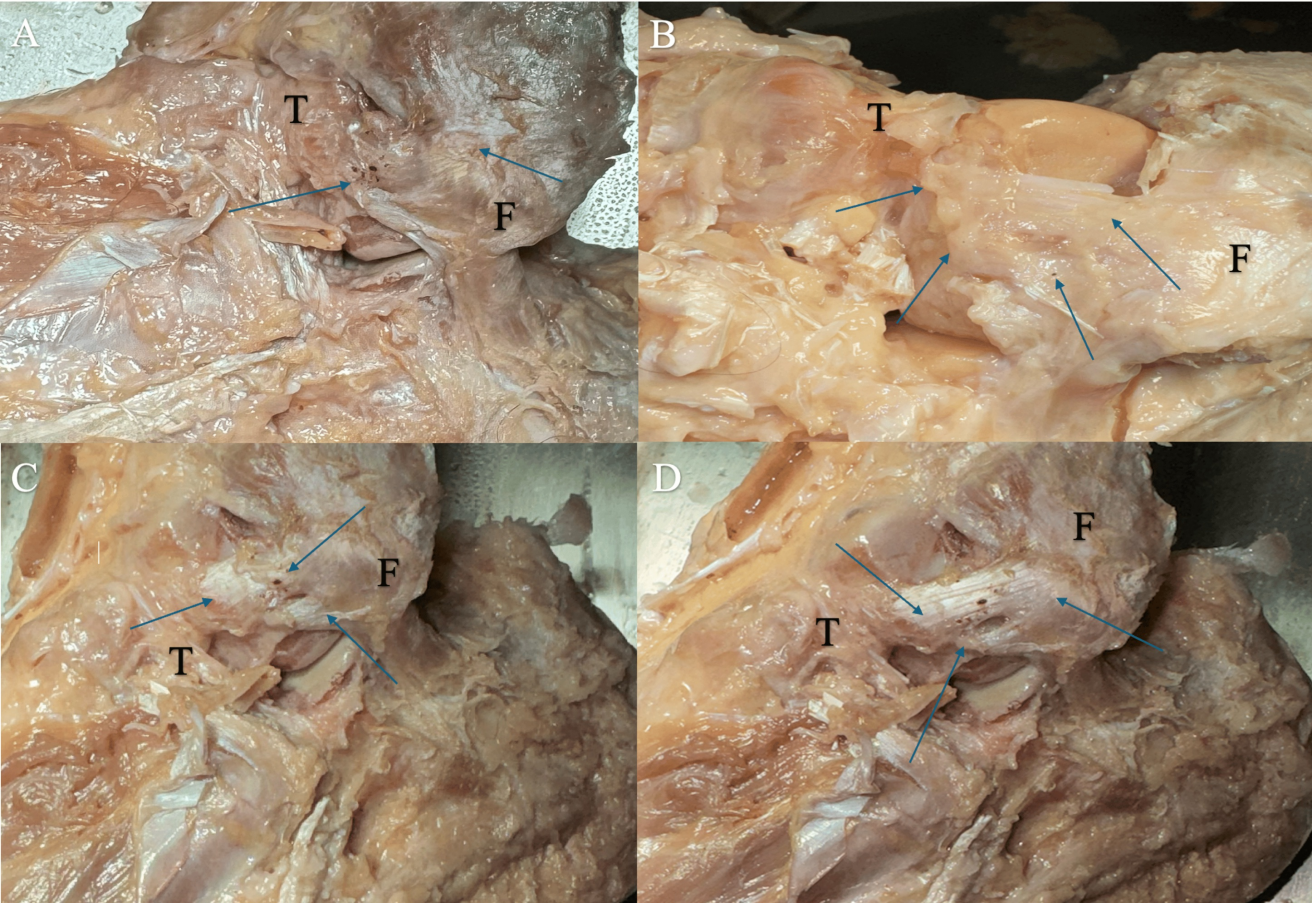
Variations of anterior tibiofibular ligament (ATFL) shapes. (A) Single-banded ATFL, (B) double-banded ATFL, (C) V-shaped ATFL, and (D) Y-shaped ATFL. Blue arrows point toward the insertion and origin points of the ligament T: talus; F: fibula

The ATFL was measured in all 22 of the ankles dissected, the CFL was inadvertently damaged in two ankles, and the PTFL was damaged in five ankles, precluding complete measurement in those specimens; these samples were excluded from the analysis. The ATFL was noted to have four different shape morphologies including single-banded (14/22 = 64%), double-banded (4/22 = 18%), V-shaped (3/22 = 14%), and Y-shaped (1/22 = 4.5%) (Figure 1). The ATFL had an average length of 23.8 mm (max = 34 mm, min = 14 mm; std: 5.61 mm) and an average width of 11.4 mm (max = 24 mm, min = 6 mm; std: 4.08 mm) (Table 1).

**Table 1:** ATFL measurements and morphology. ATFL: anterior tibiofibular ligament

Number	Length (mm)	Width (mm)	Shape
1	21	8	Single band
2	34	12	Single band
3	22	12	Single band
4	16	10	V-shaped
5	25	13	V-shaped
6	20	11	Single band
7	21	8	Single band
8 inferior	25	12	Double band
8 superior	24	7	Double band
9	14	6	Y-shaped
10	30	13	Single band
11 inferior	19	9	Double band
11 superior	16	8	Double band
12	22	7	Single band
13	26	12	Single band
14 inferior	34	12	Double band
14 superior	21	9	Double band
15	30	19	Single band
16	27	16	Single band
17	31	13	Single band
18	19	10	V-shaped
19	16	12	Single band
20 inferior	31	17	Double band
20 superior	25	24	Double band
21	23	11	Single band
22	27	7	Single band
Maximum	34	24	
Minimum	14	6	
Average	23.81	11.46	

**Figure 2:**
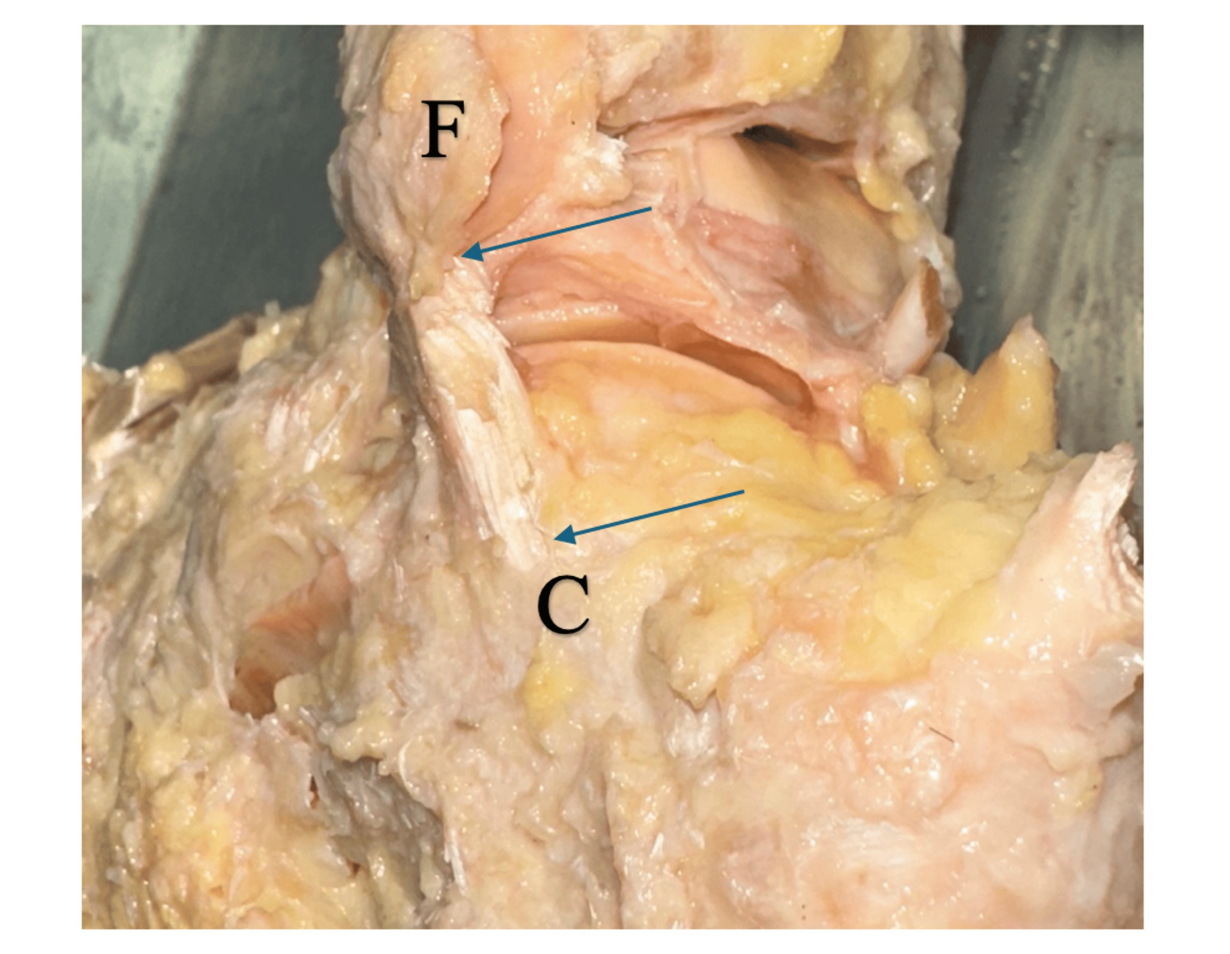
Single-banded calcaneofibular ligament. Blue arrows point toward the insertion and origin points of the ligament C: calcaneus; F: fibula

The CFL had an average length of 22.9 mm (max = 30 mm, min = 11 mm; std: 6.19 mm) and an average width of 7.8 mm (max = 12 mm, min = 3 mm; std: 2.34 mm) (Table 2). All 20 CFLs observed were in the single-band pattern (Figure 2).

**Table 2:** CFL measurements and morphology. CFL: calcaneofibular ligament

Number	Length (mm)	Width (mm)	Shape
1	12	6	Single band
2	11	3	Single band
3	26	7	Single band
4	23	10	Single band
5	23	7	Single band
6	26	7	Single band
7	20	6	Single band
8	25	9	Single band
9	26	12	Single band
10	28	7	Single band
11	26	8	Single band
12	19	9	Single band
13	26	12	Single band
14	12	6	Single band
15	29	4	Single band
16	14	7	Single band
17	25	8	Single band
18	30	10	Single band
19	30	9	Single band
20	28	10	Single band
Maximum	30	12	
Minimum	11	3	
Average	22.95	7.85	

**Figure 3:**
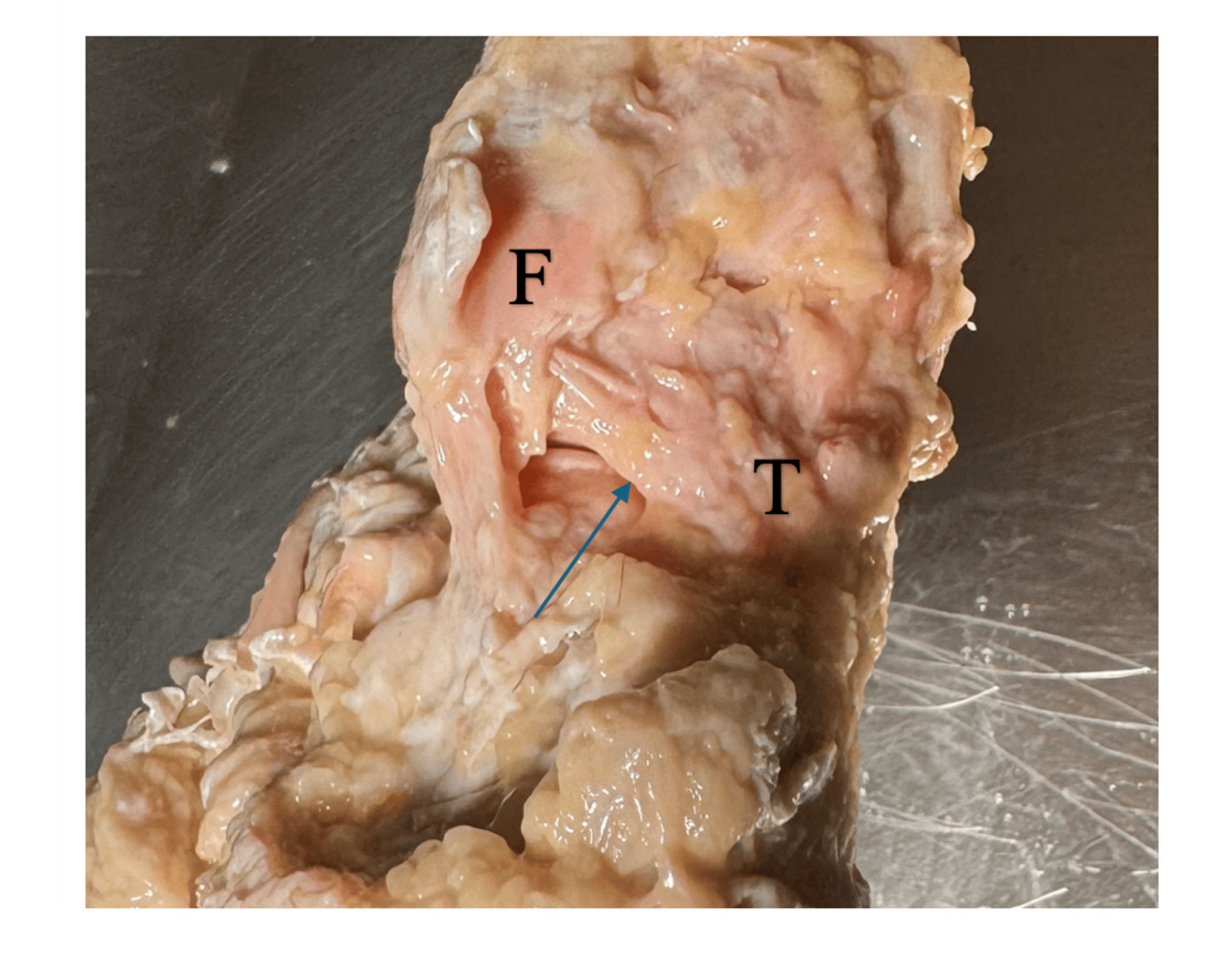
Single-banded posterior tibiofibular ligament. Blue arrows point toward the insertion and origin points of the ligament T: talus; F: fibula

The PTFL had an average length of 18.1 mm (max = 26 mm, min = 14 mm; std: 3.83 mm) and an average width of 7.8 mm (max = 13 mm, min = 2 mm; std: 2.89 mm) (Table 3). All 17 PTFLs observed were in the single-band pattern (Figure 3).

**Table 3:** PTFL measurements and morphology. PTFL: posterior tibiofibular ligament

Number	Length (mm)	Width (mm)	Shape
1	23	10	Single band
2	14	5	Single band
3	26	2	Single band
4	19	4	Single band
5	20	10	Single band
6	15	6	Single band
7	16	6	Single band
8	15	6	Single band
9	17	9	Single band
10	20	10	Single band
11	16	7	Single band
12	16	11	Single band
13	14	10	Single band
14	23	13	Single band
15	16	6	Single band
16	24	7	Single band
17	15	5	Single band
Maximum	26	13	
Minimum	14	2	
Average	18.18	7.47	

**Figure 4:**
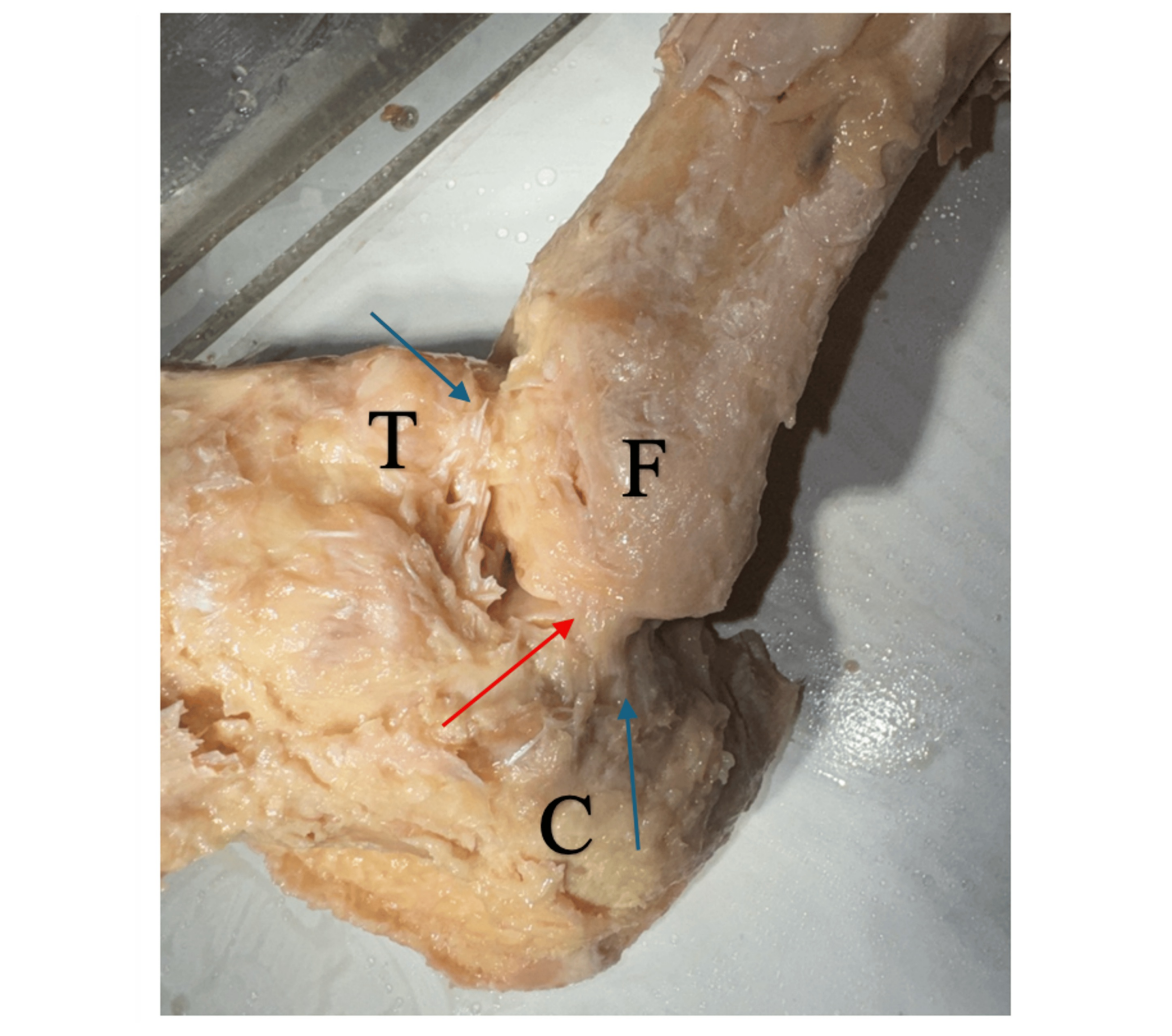
Unique fibers of the continuous ATFL to CFL. Blue arrows point toward the insertion and origin points of the ligament; the red arrow points to the continuous fibers T: talus; F: fibula; C: calcaneus; ATFL: anterior tibiofibular ligament; CFL: calcaneofibular ligament

One ankle observed had the unique morphology of having a few fibers continuous between the ATFL and CFL over the fibula (Figure 4). The length of the continuous fibers was 38 mm from the talus to the calcaneus. The widths differed in the ATFL vs. the CFL, as there were also fibers that were not continuous.

## Discussion

This cadaveric study provides valuable insights into the anatomical variations and morphometric characteristics of the lateral ankle ligaments, specifically the ATFL, CFL, and PTFL. This met the goals of this study, which was to highlight unique anatomical variation in the LALC. The findings have important implications for both clinical practice and surgical interventions related to lateral ankle instability; therefore, a precise understanding of lateral ankle ligaments is essential.

The ATFL is located on the lateral side of the ankle, connecting the lateral malleolus to the talus. It is situated perpendicular to the tibia; thus, in a supination-inversion type of ankle injury pattern, the ATFL is directly stressed and is a cause of the sprain [1,20]. It is reported to be involved in approximately 65% of all ankle-related sprain injuries; thus, a clear understanding of its anatomy is paramount [3,21]. The ATFL exhibits variability in its shape and orientation. Based on fiber arrangement, the ATFL can be classified into V-shaped, Y-shaped, parallel, single-band, and double-band (superior and inferior) configurations. Our study found that among these, the single-band shape was the most common, observed in 14 (64%) ankles. The average length measurements of the ATFL were 23.8 mm, with an average width of 11.5 mm.

Other studies have found similar variation in the ATFL anatomy. A 1994 cadaveric study on 93 ankles reported that a distinct inferior band of the ATFL was observed, but it was an inconsistent finding. They found an average length of 24.8 mm and width of 7.2 mm for the ATFL [22], but more recently, Ahmed et al. [23] found that only 53% of the ATFLs analyzed were single-banded, with a mean length of 14 mm and width of 7.6 mm. Interestingly, they noted that 73% of the ATFLs had ankle joint capsule attachments. Another study, which utilized magnetic resonance imaging (MRI) of the ankle, found that 12 of the 22 ankles imaged had double bands present [24]. Considering the potential presence of single or double bundles in the ATFL, reconstruction should address both bundles to achieve optimal functional outcomes. Recent studies demonstrated that for individuals with native double-banded ATFL structures, using modified tendon grafts for reconstruction that were separated into two distinct bundles provided improved biomechanical stability of the ankle joint [18,25]. Additionally, variations in bundle orientation, such as Y-shaped, V-shaped, or parallel, should be adapted to the specific needs of each case. Ahmed et al. [23] also noted similar findings of double-banded ATFLs in three distinct orientation patterns: V-shaped (42.86% of double bands), Y-shaped (28.57%), and parallel (28.57%). Due to individual differences in the number and orientation of bundles, obtaining MRI scans of both healthy and injured ankles can provide surgeons with valuable insights for personalized anatomical reconstruction.

The CFL runs nearly perpendicular to the ATFL and extends from the tip of the lateral malleolus of the fibula to the lateral surface of the calcaneus. Although isolated CFL tears are rare, the CFL is commonly associated with injuries to the other ligaments of the LALC [26]. This might be attributed to the fact that there are occurrences of fibers that connect both the ATFL and CFL, which were observed in one of the cadaveric ankles in this study. One study found that the inferior band of the ATFL and the CFL typically have a common fibular attachment point and are connected with arciform fibers [27]. In our study, we only observed single-banded formations of the CFL, but it has been noted that variations do exist. Other studies found the CFL in Y-shaped, V-shaped, and double-band shaped patterns in 26% to 29% of cases [23,28,29]. The arched fibers observed in the ATFL and CFL, along with their distinct banding patterns as described in the literature, could play an important role in maintaining the stability of the subtalar joint.

The PTFL is notably not a major stabilizer of the ankle joint and thus has the least incidence of injury of the three ligaments. Injury to the PTFL is rarely isolated and typically occurs concomitant with other ligament ruptures of either the ATFL/CFL or other osteochondral defects [30]. The fibers of the PTFL have varied insertions on the talus bone, and it is found to be involved in forming the tunnel of the flexor hallucis tendon [20]. Our study found that all specimens measured had a single fiber band for the PTFL. A recent cadaveric study of 12 fresh frozen donors done by Soares et al. [31] found two distinct bundles that made up the PTFL attachment to the talus, further demonstrating the need for more research in order to characterize the different morphologies of these ligaments.

Limitations of our study include that the cadavers used were embalmed with formaldehyde, which resulted in stiffness, potentially resulting in minimal measurement variations compared to living subjects. This phenomenon of cadaveric studies embalmed in formaldehyde is noted previously in the literature; one study showed an approximate 14% increase in axial and torsional stiffness of the diaphyseal bones, and another study showed a decrease between 80% and 96% in range of motion of the spinal column [32,33]. Furthermore, formaldehyde fixation has been associated with changes in tissue morphology. In fact, one study demonstrated that cutaneous surgical specimens exhibited a mean reduction in length of 17.0% and width of 9.5% between excision and completion of formalin fixation [34]. Additionally, the embalmed cadavers limited the level of dissection that could be performed as the ankle tissue began to lose its integrity, making it difficult to discriminate certain structures from the LALC. Lastly, the study was limited by the number of ankles available for examination, as the cadavers were obtained from the medical school anatomy laboratory. This also limited our ability to compare variations between contralateral ankles from the same cadaver, which may have provided additional insight into anatomic variation. A multicenter study with a larger sample size would be ideal in providing more robust and generalizable data, thereby improving the validity and applicability of our findings to a wider population.

## Conclusions

In conclusion, these results highlight the variability of the LALC in both size and shape. The variability found in the anatomy of the LALC challenges the current simplistic view of the structures of the ligament complex. A thorough understanding of LALC anatomy can enable surgeons to make informed decisions during ligament reconstruction, potentially leading to improved graft placement, enhanced ankle stability, and reduced long-term complication rates. This study also warrants future research with direct biomechanical or clinical correlation studies to further strengthen how the differences in LALC anatomy and morphology impact the clinical setting.
